# The association between coping method and distress in infertile woman: A cross-sectional study from Turkey

**DOI:** 10.12669/pjms.316.8605

**Published:** 2015

**Authors:** Hande Dag, Sayime Yigitoglu, Belgin Iyik Aksakal, Oya Kavlak

**Affiliations:** 1 Dr. Hande Dag, PhD. Research Assistant, Adnan Menderes University, School of Health, Women’s Health and Diseases Nursing Department, Aydin, Turkey; 2 Sayime Yigitoglu, Msc. the Family Planning and Infertility Application & Research Center, Ege University Hospital, Izmir, Turkey; 3 Belgin Iyik Aksakal, RN. the Family Planning and Infertility Application & Research Center, Ege University Hospital, Izmir, Turkey; 4 Oya Kavlak, Associate Professor, Women’s Health and Diseases Nursing Department, Ege University Faculty of Nursing, Izmir, Turkey

**Keywords:** Coping behaviour, Psychological stress, Reproductive sterility

## Abstract

**Objectives:**

To assess the distress level in infertile women and their coping skills.

**Methods:**

One hundred and twenty-seven infertile women who had been referred to the Family Planning and Infertility Research and Practice Center (IRPC) of a university hospital to receive therapy between June 2012-2013 were enrolled in this study. Several surveys, including the “Infertile Woman Identification Form”, the “Infertility Distress Scale (IDS)” and the “Ways of Coping Inventory (WCI),” were used as data collection tools.

**Results:**

The mean age of the women who participated in the study was 32.34 ± 5.44. They had been on therapy for 3.95 ± 3.21 years and had been referred for therapy 2.73 ± 1.76 times. The mean score of the IDS was determined to be 37.0 ± 9.7 (23–66), and the mean score of the WCI subscale was 1.86 ± 0.55 (0.5-3.0). In the IDS and WCI subscales, statistically significant negative relationships were detected between “Optimism” (r=-0.327), “Seeking Social Support” (r=-0.255), and “Self-Confidence” (r=-0.305), whereas there were statistically significant positive relationships between “Helplessness” (r=0.376) and “Submissiveness” (r=0.278) (p<0.01).

**Conclusion:**

The women who developed negative coping strategies had higher infertility distress scores than other women.

## INTRODUCTION

According to the WHO’s reports, the number of couples affected by infertility in 1990 were 42 million which increased to 48 million in 2010 as a result of population growth.^1^,^2^ In Turkey, the infertility rate was determined to be 9.4% by the Turkish Demographic and Health Survey.^3^ Infertility presents itself as a sudden or unanticipated life crisis and sometimes cannot be explained. Its diagnosis is often prolonged, which causes extreme stress and coerces concordance mechanisms.^1^,^4^ Various stressors, such as a woman’s motherly instinct and the value given to reproductive woman by society, can affect infertile women negatively.^3^,^5^ It has been reported that a vast majority of childless women divorce their spouses, disrupt their relationships with couples who have children, have problems with health insurance politics, and develop anxiety and depression disorders.^6^,^7^

Most studies of women influenced by infertility have focused on stressors, culture, relationships, anxiety and depression levels, the treatment process, or health politics.^4^,^8^-^10^ In particular, two studies that used the Infertility Distress Scale (IDS) found that women were more influenced by infertility with increasing age, education level, marital duration, time desiring a child.^10^,^11^ However, these studies were carried out in the Central Anatolia region of Turkey, and there are cultural, financial and educational differences between this and other regions within Turkey. These studies concluded that to increase the success of infertility treatment, there is a need for increasing health professionals’ commitment to understanding infertile women. It is vital to gain psychological information from them about the patients influenced by infertility in Izmir, in the Aegean Region of Turkey.

The aim of this research was to study the distress level in infertile women and their coping skills.

## METHODS

This cross-sectional study was carried out at a Family Planning and Infertility Research and Practice Center (IRPC) at a university hospital in Izmir, which is the third largest city in western Turkey. IRPC accepts patients from all over the country and is the first and only state infertility centre.

Infertile women who voluntarily sought treatment and who were for the most part literate and lacking any chronic or psychiatric disease were referred to IRPC to receive therapy between June 2012 to 2013 and were selected through a random sampling method. The sample size of the study was calculated using a pre-established formula using TDHS’s infertility rate of 2003 as the basis: n= (t^2x^p^x^q)/(d^2^).^3^

In this research, 131 women, whose mean age was 32.3 ± 5.4 years, were targeted; however, four women were excluded from the study because they completed the questionnaire incorrectly. Therefore, the study was carried out on 127 subjects. As data collection tools, the “Infertile Woman Identification Form (IWIF)”, the “Infertility Distress Scale (IDS)” and the “Ways of Coping Inventory (WCI)” were used. Face to face interviews were performed with each participant privately in a room of the IRPC unit when they came for a treatment appointment.

The IWIF was developed to determine infertile women’s sociodemographic features, including age, educational level, occupation, income status, descriptive data related to the diagnosis and therapy process, environmental factors, and ability to cope with stress.

The IDS was developed by Akyuz et al.^11^ to determine the level of the psychological effect of infertility and the therapy process on Turkish women. In this scale, the Cronbach’s alpha value of the item scores was determined to be 0.93. Statements are used to describe an individual’s emotional status, and placed opposite the statements are boxes to indicate the incidence of each emotion. The IDS comprises a total of 21 items, 16 of which are positive and 5 are negative (items 3, 10, 13, 14 and 21 are the negative statements). Responses were gauged by a Likert scale (1=never to 4=always). The negative statements are scored reversely. The scale does not contain any subgroups, and the lowest score that can be obtained is 21, while the highest is 84. A higher score implies that the effect of infertility is high.^11^ The scale does not have a cut-off for determining the level of infertility distress.

In this study, the Cronbach’s alpha value of IDS was determined to be 0.85. The Cronbach’s alpha values calculated fell within acceptable limits.

The WCI was developed by Folkman and Lazarus (1980) and was adapted into Turkish by Sahin and Durak (1995).^12^,^13^ This scale has two dimensions: ways of coping with problems and ways of coping with emotions. These two dimensions are reflected by five subscales, which consist of seven items related to “Self-Confidence” (8, 10, 14, 16, 20, 23, 26), five items related to “Optimism” (2, 4, 6, 12, 18), eight items related to “Helplessness” (3, 7, 11, 19, 22, 25, 27, 28), six items related to “Submissiveness” (5, 13, 15, 17, 21, 24), and 4 items related to “Seeking Social Support” (1, 9, 29, 30). The Confidence Coefficients of these subscales are .80, .68, .73, .70, and .47, respectively.^13^ In this study, they were determined to be .84, .77, .79, .61, and .36, respectively.

In this scale, which comprises a total of 30 items (scoring between 0-3), the items 1 and 9 are calculated by scoring reversely during the estimation of the “Seeking Social Support” factor. The scores belonging to each factor are calculated separately and obtained from the questions belonging to each factor, and the sum of that factor is divided by the number of questions. A mean score related to that factor is then obtained. The total score cannot be calculated.

Statistical analyses were performed using the Statistical Package Program for Social Sciences, version 18.0 (SPSS, Inc., Chicago, IL). One-Way ANOVA, the Mann-Whitney U Test and Kruskal Wallis correlation and Tukey statistical tests were used to analyse the research findings as appropriate.

The necessary written approvals were obtained from the Scientific Ethic Committee of Nursing Faculty (2012-33), the IRPC of the university hospital where the research was carried out, and from the participants and the owners of the scales.

## RESULTS

Participants had been undergoing therapy for 3.95 ± 3.21 years and had been referred for therapy 2.73 ± 1.76 times. Of them, 40.2% had graduated from a university and 29.1% had received insemination and *in vitro* fertilization (IVF) therapy.

72.4% of the women found both the diagnosis and therapy processes stressful, and 81.9% had received information related to this process, 51.2% of whom had received that information from nurses. A majority of women (70.9%) thought that the health professionals’ approaches were very good, understanding, and good-humoured; 15% felt that the professionals should have been more understanding and informative, and 11.8% felt that their treatment was normal. However, 2.4% were satisfied with the nurses, but evaluated the doctors as non-informative.

The mean of total IDS scores was determined to be 37.0 ± 9.7 (23-66). The variables which were affecting the mean IDS score are presented in Table-I. Women who perceived their income status to be good (x=32.10) or bad (x=41.45) and had received a university (x=34.11) and elementary school education (x=40.69) scored higher on the IDS and were more affected by infertility than others (p< 0.05). The women who received therapy four or more times (x=43.13) or who were unable to receive information related to therapy (x=43.15) found the health professionals’ approaches normal (x=43.20) and those who were satisfied with the nurses but did not receive sufficient information from the doctors (x=25.66) scored higher on the IDS and were highly affected by infertility (p<0.05).

**Table-I:** Variables Affecting Mean IDS Score.

	Mean Scores of IDS				
Variables	N	Mean ± Sd	Test Value KW/U	df	P
*Income Status*					
300 € and less	41	38.4 ± 10.0	8.887^+^	3	0.03*
301 €- 565 €	40	39.2 ± 9.6			
566 €- 847 €	28	34.3 ± 8.5			
848 € and more	18	33.4 ± 9.6			
*Education*					
Literate	6	44.8 ± 8.1			
Primary school	42	40.6 ± 10.0	17.597^+^	4	0.001*
High school	26	35.8 ± 9.0			
University	51	34.1 ± 8.8			
Illiterate	2	28.5 ± 3.5			
*Receiving Therapy*					
1 time	32	35.4 ± 9.1	14.893^+^	3	0.002*
2 times	36	35.2 ± 9.9			
3 times	29	34.8 ± 7.8			
4 and more times	30	43.1 ± 9.8			
*Getting Information About Therapy*					
Received	104	36.0 ± 9.1	6.605^+^	2	0.03*
Not received	19	43.1 ± 11.6			
Finding not enough	4	34.5 ± 3.4			
*Approach of Health Staff*					
Very nice	90	36.3 ± 9.5	12.093^+^	3	0.007*
Normal	15	43.2 ± 9.5			
Satisfied with nurse, doctors don’t provide information	3	25.6 ± 1.5			
More understanding and informative	19	37.3 ± 9.3			
*Finding Therapy and Diagnosing Process Stressful*					
Find stressful	92	38.5 ± 10.0	1105.50^x^		0.008*
Not find stressful	35	33.2 ± 7.8			
Total	127				

* p<0.05,

+ compared with Kruskal-Wallis test,

x compared with U=Mann-Whitney U test

The mean scores of the WCI subscales were determined as shown in Table-II. The variables which were affecting the WCI subscales are presented in Table-III. As a result of the advanced analysis performed, we identified a significant difference in the WCI’s “Helplessness” subscale in women for whom 7-9 years had elapsed while waiting to have a baby (x=1.86) and the group which had received therapy between 2 (x=1.29) and 4 (x=1.76) years of waiting, as well as between those with a primary education (x=1.67) and higher education graduates (x=1.24) (p<0.05).

**Table-II:** Mean Scores of the WCI.

WCI Subscales	The Importance of Education Needs of Women	Lowest and Highest Values
	Mean ± SD (Min-max)	
Self- confidence	2.29± 0.59 (0.71-3.0)	0 - 3
Optimism	2.07± 0.62 (0.4-3.0)	
Helplessness	1.45± 0.68 (0.0-3.0)	
Submissiveness	1.50± 0.62 (0.3-3.0)	
Seeking social support	1.86± 0.55 (0.5-3.0)	

**Table-III:** Variables Affecting WCI Subscales.

Variables	Self- Confidence	Optimism	Helplessness	Submissiveness	Seeking Social Support
*Expected Time for Having a Child*					
1 - 3 years	2.2 ± 0.5	1.9 ± 0.6	1.3 ± 0.5	1.3 ± 0.5	1.7 ± 0.4
4 - 6 years	2.4 ± 0.5	2.1 ± 0.5	1.3 ± 0.7	1.5 ± 0.6	1.8 ±.06
7 - 9 years	2.1 ± 0.6	2.0 ± 0.5	1.8 ± 0.6	1.7 ± 0.6	2.0 ± 0.6
10 years and more	2.2 ± 0.5	2.0 ± 0.6	1.5 ± 0.6	1.5 ± .07	1.8 ±.05
KW (p)	7.711 (0.10)	2.879 (0.41)	11.946 (0.00*)	8.075 (0.04*)	2.566 (0.46)
*Education*					
Illiterate	2.7 ± 0.5	2.7 ± 0.1	2.5 ± 0.5	2.0 ± 0.1	2.2 ± 0.0
Literate	2.3 ± 0.6	2.3 ± 0.6	1.9 ± 0.9	1.7 ± 0.9	1.6 ± 0.5
Primary school	2.0 ± 0.4	2.0 ± 0.6	1.6 ± 0.6	1.7 ± 0.6	1.7 ± 0.6
High school	2.1 ± 0.5	2.1 ± 0.5	1.3 ± 0.6	1.3 ± 0.5	1.8 ± 0.5
University	2.0 ± 0.5	2.0 ± 0.6	1.2 ± 0.5	1.3 ± 0.5	1.9 ± 0.5
KW (p)	2.759 (0.43)	3.332 (0.50)	14.435 (0.00*)	12.650 (0.01*)	6.477 (0.16)
*Receiving Treatment*					
1 time	2.2 ± 0.6	1.8 ± 0.6	1.4 ± 0.6	1.5 ± 0.5	1.8 ± 0.5
2 times	2.2 ± 0.6	2.1 ± 0.5	1.2 ± 0.7	1.4 ± 0.7	1.8 ± 0.5
3 times	2.2 ± 0.5	2.0 ± 0.6	1.3 ± 0.5	1.3 ± 0.5	1.9 ± 0.5
4 and more times	2.3 ± 0.5	2.1 ± 0.5	1.7 ± 0.6	1.7 ± 0.5	1.8 ± 0.6
KW (p)	1.343 (0.71)	2.847 (0.41)	8.666 (0.03*)	7.972 (0.04*)	0.480 (0.92)
*Getting Information About Treatment*					
Received	2.3 ± 0.5	2.0 ± 0.6	1.3 ± 0.6	1.4 ± 0.5	1.9 ± 0.5
Not received	2.1 ± 0.6	2.1 ± 0.5	1.7 ± 0.8	1.8 ± 0.8	1.5 ± 0.5
Finding not enough	2.3 ± 0.5	1.9 ± 0.5	1.8 ± 0.8	1.5 ± 0.4	2.1 ± 0.2
KW (p)	1.086 (0.58)	0.121 (0.94)	2.763 (0.25)	4.951 (0.08)	8.858(0.01*)
*Source of Information*					
Nurse	2.2 ± 0.6	2.0 ± 0.6	1.5 ± 0.6	1.5 ± 0.6	1.8 ± 0.5
Doctor	2.5 ± 0.4	2.2 ± 0.5	1.1 ± 0.4	1.4 ± 0.4	2.1 ± 0.4
Other	2.1 ± 0.5	1.9 ± 0.5	1.4 ± 0.7	1.5 ± 0.6	1.8 ± 0.6
KW (p)	6.231 (0.04*)	4.945 (0.08)	4.646 (0.09)	0.485 (0.78)	4.129 (0.12)
*Approach of Health Staff*					
Very good	2.3 ± 0.5	2.1 ± 0.5	1.4 ± 0.6	1.5 ± 0.6	1.8 ± 0.5
Normal	1.9 ± 0.6	1.7 ± 0.5	1.6 ± 0.7	1.7 ± 0.6	1.8 ± 0.6
Satisfied with nurse, not doctors	2.8 ± 0.2	2.7 ± 0.4	0.7 ± 0.3	1.1 ± 0.2	2.3 ± 0.5
More Understanding and informative	2.1 ± 0.5	1.7 ± 0.6	1.4 ± 0.6	1.3 ± 0.6	1.8 ± 0.4
KW (p)	11.001 (0.01*)	20.208 (0.00*)	6.367 (0.09)	2.987(0.39)	3.969 (0.26)

KW: Kruskall Wallis,

* p < 0.05

The mean scores of the WCI’s “Self-Confidence” subscale was found to be higher in women who had received information from doctors (x=2.59) (F=3.03, p=0.05) and in those who had found the health workers’ approaches very good (x=2.36) (F=3.79, p=0.01). The “Submissiveness” subscale was found to score higher in women who had received therapy four or more times (x=1.76) (F=2.71, p=0.04), who had been trying to conceive for 7-9 (x=1.73) and 1-3 (x=1.31) years (F=2.74, p=0.04) or who were graduates of either elementary (x=1.73) or higher (x=1.33) education (F=3.43, p=0.01). The “Seeking Social Support” subscale score was found to be lower in women who were unable to receive information (x=1.52) compared to the others (F=4.74, p=0.01). The “Optimism” subscale was found to be higher among women who had evaluated the health workers’ approaches as very good (x=2.18) and who were satisfied with the nurses but had not received information from doctors (x=2.73) (F=6.77, p=0.00).

From the correlation analysis performed between the IDS and WCI subscales (Table-IV), we identified a statistically significant negative relationship between optimism (r=-0.327), seeking social support (r=-0.255) and self-confidence (r=-0.305), and a positive relationship between helplessness (r=0.376) and submissiveness (r=0.278) (p<0.01).

**Table-IV:** Correlation Analysis Results Between the IDS and WCI Subscales Mean Scores.

WCI Subscales Mean Scores	IDS Mean Scores		
	N	r^+^	p
Self- confidence	127	- 0.305	0.00 *
Optimism		- 0.327	0.00 *
Helplessness		0.376	0.00 *
Submissiveness		0.278	0.002 *
Seeking social support		- 0.255	0.004 *

r: Correlation Test,

* p < 0.01

## DISCUSSION

The mean scores of the IDS determined in our study were similar to those previously reported.^10^,^14^,^15^ In studies similar to ours, increasing length of the marriage and education level affected the mean score of the IDS.^10^,^11^,^16^ Although it has been reported in other studies, we did not identify a statistically significant relationship between the length of time which had elapsed while trying to conceive, the length of therapy, age, social security, employment status, support during the process of diagnosis and therapy, alterations in familial behaviours, and the mean IDS score.^15^,^16^ Moreover, in contrast to other studies, we observed that the number of therapy sessions, the perception of income status, information received related to the length of therapy, and health workers’ approaches did affect the scores obtained from the IDS in our study.

In this study, as in Unal et al.’s study, women who perceived their income as good or bad were more influenced by infertility compared to those who perceived it as normal.^10^ In other studies, the financial burden of therapy impaired childless women’s mental health, indicating that the level of depression, psychological distress, and loneliness is reduced as the level of income increases.^17^,^18^ In addition, an important relationship has been reported between the money spent throughout the process of infertility therapy and the stress level of the women undergoing therapy.^19^

Furthermore, it was determined that women generally received information related to the infertility process from nurses and were content with the approaches of these health workers. Nurses provide health care to infertile couples more frequently than other health professionals, and should understand the couple’s emotional status to better help them.^20^,^21^

Infertility can lead to sexual dysfunction, depression, anxiety and relationship problems, and without sufficient patient education regarding the therapy process, can increase the stress levels and affect the coping skills of the women undergoing the therapy. Additionally, in other studies, it was observed that passive avoidance has been used by women with elevated stress levels to cope with stress. Consequently, as stress increases, avoidance behaviour towards problems, responsibilities and seeking social support results in a decrease in logical problem solving.^10^,^22^,^23^,^24^ Individuals exhibit physical and emotional responses to stressors. If control is not established, these individuals might get worse and become emotionally isolated. This may result in a lack of treatment. All of these factors can negatively impact infertile individually, thereby causing women to discontinue treatment or to fail at treatments that they pursue.^4^,^5^,^25^

### Limitations of the Research

Due to the nature of the IDS used, only women participated in the sampling, there was no control group, and different levels of infertility (i.e., early diagnosis, tests, therapy failure, or abortion) were included and given equal weight. At the end of this study, it was determined that women who developed positive coping strategies were less influenced by infertility, but those who developed negative coping strategies were influenced more. Consequently, additional studies and attempts to help infertile women referred to infertility clinics to develop positive coping strategies are recommended.
